# High temperature increased lignin contents of poplar (*Populu*s spp) stem *via* inducing the synthesis caffeate and coniferaldehyde

**DOI:** 10.3389/fgene.2022.1007513

**Published:** 2022-09-09

**Authors:** Xianyan Zhao, Panpan Li, Xingwang Liu, Tianyu Xu, Yuqing Zhang, Haifeng Meng, Tao Xia

**Affiliations:** ^1^ State Key Laboratory of Biobased Material and Green Papermaking, School of Bioengineering, Qilu University of Technology, Jinan, China; ^2^ Tai’an Forestry Protection and Development Center, Tai’an, China

**Keywords:** high temperature, lignin metabolic intermediates, PtrMYBs, PtrC3'H1, PtrCCR2, transcript and metabolic profiling

## Abstract

Lignin contributes to plant resistance to biotic and abiotic stresses and is dominantly regulated by enzymes which catalyze the generation of metabolites intermediates in lignin synthesis. However, the response of lignin and its key regulatory factors to high temperature stress are poorly understood. Here, this finding revealed that the content of lignin in poplar (*Populu*s spp) stem increased after 3 days of high temperature stress treatment. In fourteen metabolic intermediates of lignin biosynthetic pathway with targeted metabolomics analysis, caffeate and coniferaldehyde increased evidently upon heat stress. C3’H (*p*-Coumaroylshikimate 3-hydroxylase) and CCR (Cinnamoyl-CoA reductase) are recognized to catalyze the formation of caffeate and coniferaldehyde, respectively. Transcriptome data and RT-qPCR (reverse transcription-quantitative real-time polymerase chain reaction) analysis uncovered the high transcriptional level of *PtrMYBs* (*PtrMYB021*, *PtrMYB074*, *PtrMYB85*, *PtrMYB46*), *PtrC3’H1* (Potri.006G033300) and *PtrCCR2* (Potri.003G181400), suggesting that they played the vital role in the increase of lignin and its metabolic intermediates were induced by high temperature. The discovery of key regulators and metabolic intermediates in lignin pathway that respond to high temperature provides a theoretical basis for quality improvement of lignin and the application of forest resources.

## Introduction

Forest resources not only have great application value for vegetation restoration, soil erosion prevention and saline-alkali land restoration, but also can be used for the development of biomass and fiber energy (Nakahara et al., 2019). Lignin plays a vital role in cell wall formation, especially in wood and bark, and it permits the xylem to maintain a certain amount of water transport, mechanical support, growth and development for plants (Zhao and Dixon, 2011). However, lignin is also a major obstacle to the utilization of forest resources (Van Acker et al., 2013; Behr et al., 2019). Therefore, the study of lignin is of great significance to the utilization of forest resources.

High temperature stress is an important factor affecting plant growth and development (Zhu, 2016). And lignin is essential in plant response to stresses such as high temperature stimuli (Cesarino, 2019). Plant stems are subjected to rapid lignification of cell walls with high temperatures treatment (Crivellaro and Büntgen, 2020). During lignification, lignin accumulation is deposited in the cell wall, which enhances the stiffness of the cell wall and makes the xylem cell wall less permeable to water, facilitating the long-distance transport of water, minerals, and organic matter in the plant (Zhao, 2016). Plants tend to synthesize more lignin so as to enhance their resistance to high temperature stress.

Lignin synthesis is a complex network, including the lignin monomers biosynthesis, transport and polymerization process (Vanholme et al., 2019). Lignin monomers are synthesized in the cytoplasm through a series of reactions including hydroxylation, deamination, reduction, methylation and transportation to the apoplast (Vanholme et al., 2019). Seconldly, lignin is produced by the polymerization of lignin monomers in the secondary cell wall (Vanholme et al., 2008; Bonawitz and Chapple, 2010; Miao and Liu, 2010; Vanholme et al., 2019). According to the differences in lignin monomers and crosslinking methods, lignin monomers can be classified as three main forms, including p-hydroxyphenyl (H), guaiacyl (G), and syringyl (S) (Vanholme et al., 2008; Vanholme et al., 2019).

Many enzymes are involved in formation of lignin monomer, and the expression level of the genes encoding these enzymes directly affects the content and deposition of lignin (Vanholme et al., 2008; Vanholme et al., 2019). Lignin is polymerized from the monomers of phenylpropane derivatives. Recently, researches showed that the change of phenylpropanoid biosynthetic enzymes in transgenic poplar had great influence on lignin content (Wang et al., 2018). Phenylalanine ammonia-lyase (PAL), cinnamate 4-hydroxylase (C4H), and 4-coumarate CoA ligase (4CL) are three enzymes which are related with the first three catalytic reactions of the traditional phenylpropane pathway, respectively (Rohde et al., 2004; Chen et al., 2006; Vanholme et al., 2008; Wang et al., 2018). The decline of PAL, C4H and 4CL expression reduced the lignin content (Rohde et al., 2004; Chen et al., 2006; Vanholme et al., 2008; Wang et al., 2018). Many other enzymes such as quinate/shikimate p-hydroxycinnamoyltransferase (HCT), p-coumaroylshikimate 3-hydroxylase (C3’H), caffeoyl shikimate esterase (CSE), caffeic acid O-methyltransferase (COMT), caffeoyl-CoA O-methyltransferase (CCoAOMT), cinnamoyl-CoA reductase (CCR), ferulate 5-hydroxylase (F5H), and cinnamyl alcohol dehydrogenase (CAD), are essential to maintain a normal level of lignin content (Barros et al., 2015; Xie et al., 2018). The absence of any enzyme more or less affects the synthesis of lignin (Barros et al., 2015; Xie et al., 2018).

C3’H is a phenolic enzyme that catalyzes the production of p-coumaric acid to caffeic acid and p-coumaryl shikimic acid to catechylshikimic acid. The expression level of *C3’H* directly affects the lignin content and S/G ratio (Coleman et al., 2008; Bryant et al., 2020). *CYP98A3* gene was isolated in early bioinformatics experiments, which may encode C3’H enzyme in *Arabidopsis* (Schoch et al., 2001). In alfalfa, the content of lignin in transgenic plants with increased *C3’H* expression was 5% higher than that in wild-type plants (Reddy et al., 2005; Pu et al., 2009). The lignin content in *P. alba × P. grandidentata* reduced significantly when the expression of *C3’H* was inhibited (Coleman et al., 2008; Peng et al., 2021). Although the reduction of *C3’H* expression will lead to dwarfed phenotype, relevant studies have shown that the specific components in mediator complex can contribute to the normal growth of plants and the increase of H-type lignin, which is conducive to the conversion of bioenergy (Bonawitz et al., 2014).

CCR catalyzes the generation of hydroxycinnamaldehydes from hydroxycinnamoyl-CoA esters, and its down-regulation causes the decline of lignin content (Ruel et al., 2009; Van Acker et al., 2014). *ccr1* mutant in maize changed the lignin deposition in the walls of the sclerenchymatic fibre cells surrounding the vascular bundles and lignin content (Tamasloukht et al., 2011). Besides, reduction of lignin caused by decreased *CCR* expression brings about lower ferulic acid resulting in a decrease in polymers (Leplé et al., 2007; Ralph et al., 2008). Down-regulation of *CCR* gene expression can reduce the conversion of poplar into ethanol and even the biomass of poplar (Van Acker et al., 2014). A recent study found that *CCR2* knockdown can reduce lignin content in poplar trees without affecting their normal growth (De Meester et al., 2020). Besides, the overexpression of *OsCCR* can increase the lignin content and enhance the resistance to pathogenic bacteria in rice (Kawasaki et al., 2006).

COMT is the methylation enzyme of lignin biosynthesis, which is responsible for the methylation of lignin precursor (Daly et al., 2019). It catalyzes the methylation of a number of substances such as 5-hydroxy coniferyl alcohols, caffeoyl, free acids and aldehydes (Daly et al., 2019). Inhibition of *COMT* transcription level in tobacco and poplar decreased lignin content (Dwivedi et al., 1994; Jouanin et al., 2000). In ryegrass, the biomass and phenotype unchanged with *COMT1* expression disturbed (Tu et al., 2010). Significantly, the economic value of ryegrass was increased by the reduction of lignin and its components (Tu et al., 2010). The transcriptional level and enzyme activity of COMT can directly or indirectly affect the production of lignin, coumarin, flavonoids, organic acids and other metabolites (Saluja et al., 2021). Therefore, *COMT* may play a key role in promoting the growth and development of plant and its adaptation to the environment, as well as in the synthesis of ferulic acid.

NAC and MYB transcription factors (TFs) are the two most important classes of TFs that regulate lignin (Ohtani and Demura, 2019). NAC TFs PtrVND6 and PtrSND1 regulate lignin synthesis involved in poplar growth and development (Lin et al., 2017). Downstream of NAC TFs, MYB TFs such as AtMYB46 and AtMYB83 are involved in lignin synthesis by regulating the expression of some structural genes in the lignin synthesis pathway (Ohtani and Demura, 2019). Thus, it is crucial to explore the involvement of environmental factors in lignin synthesis through NAC and MYB TF.

Poplar (*Populu*s spp), which is an important forest resource, is served as a model material to study the basic biological characters of trees except for shelter forest, road greening and papermaking (Taylor, 2002). With the intensification of human activities, the occurrence of extreme weather is increasingly frequent. Therefore, it is of great significance for the utilization of bioenergy to understand the molecular mechanism of lignin response to extreme weather such as high temperature and explore candidate genes related to lignin synthesis and metabolism in response to high temperature stress in poplar.

## Materials and methods

### Plant material and experimental conditions

Poplar tissue culture plantlets “84k” were presented by Lingli Li of Northwest A&F University and cultured under 25°C and 16/8 h long-day condition. Tissue culture plantlets for about 40 days old were placed in an incubator with 35°C treatment, and sampled at 0, 6, 12, 24, 48, and 72 h, respectively. Samples were taken at 0 and 72 h after treatment at 25°C, respectively. The collected samples were quickly placed in liquid nitrogen and then stored in an ultra-cold refrigerator at -80°C.

### Determination of lignin content

Lignin content was determined according to the kit description (Suzhou Comin Bioltechnology Co., Ltd, Suzhou, China). The sample was dried at 80°C to constant weight, crushed, passed through a 40-mesh sieve, and weighed out about 5 mg (denoted as W) in a 10 ml glass test tube. Add 1 ml bromoacetyl-glacial acetic acid and 40 μL perchloric acid to the blank tube and sample tube, sealed the glass test tube with sealing film, mixed well, bathed in water at 80°C for 40 min, shaked every 10 min, then cooled naturally. Added 1 ml NaOH-acetic acid solution to glass test tube and mixed thoroughly. Took 40 μL supernatant and added 1960 μL glacial acetic acid (adjusted the amount of glacial acetic acid properly to ensure the absorbance value between 0.1–0.8) and mixed well. 1 ml of the mixed sample was placed in A quartz colorimetric dish to determine the absorbance value A at 280 nm. They were denoted as blank tube A and measuring tube A respectively. △A = A measuring tube -A blank tube.

$$\text{Lignin}\;\left(\text{mg}/g\;\text{dry}\;\text{weight}\right)={\left(\Delta A-0.0068\right)\;/0.0694*V\;*10}^{-3}/W*T$$

$$={\left(\Delta A-0.0068\right)\;}^{*}{0.0294/W}^{*}T$$

V: total volume of reaction, 2.04 ml; W: sample mass, g; T: dilution multiple.

### Extraction and collection conditions of intermediate metabolites of lignin

Measurement of intermediate metabolites of lignin was conducted by Metware Company with Ultra Performance Liquid Chromatography and Tandem Mass Spectrometry (UPLC-MS/MS). Samples were extracted as follows: biological samples were vacuum freeze-dried and ground (30 Hz, 1.5 min) to powder form using a grinder (MM 400, Retsch). The 100 mg of powder was weighed and dissolved in 0.6 ml of extraction solution, then the dissolved sample was placed in a refrigerator at 4°C for 12 h. After centrifugation (10, 000 g for 10 min), the supernatant was aspirated and the sample was filtered through a microporous membrane (0.22 μm pore size) and stored in the injection vial for UPLC-MS/MS analysis.

UPLC-MS/MS analysis contained liquid phase and mass spectrometry conditions. The specific steps and methods of the liquid phase were as follows: 1) Chromatographic column: Waters ACQUITY UPLC HSS T3 C18 1.8 µm, 2.1 mm*100 mm; 2) mobile phase: ultra-pure water (with 0.04% acetic acid) for phase A and acetonitrile (with 0.04% acetic acid) for phase B; 3) elution gradient: 5% for phase B at 0.00 min, linearly increasing to 95% for phase B at 10.00 min and maintaining at 95% for 1 min, decreasing to 5% for phase B from 11.00 to 11.10 min, and balanced at 5% until 14min; 4) Flow rate 0.35 ml/min; column temperature 40 °C; injection volume 4 μL. Mass spectrometry conditions were as follows: electrospray ionization (ESI) at 550 °C, mass spectrometry voltage 5500 V, curtain gas (CUR) was 30 psi, collision-activated dissociation (CAD) was high, declustering potential (DP) was optimized, and collision energy (CE) was scanned for detection (Chen et al., 2013).

The conditions and parameters of mass spectrometry were shown below: electrospray ionized (ESI) source temperature was 550 °C, CURTAIN gas was 30 PSI, mass spectrometry voltage was 5500 V, along with high collisions activate dissociation, dissociation potential optimization, and collision energy scanning.

### Qualitative and quantitative analysis of metabolites

Based on the self-built database MWDB (metware database) and the public database of metabolite information, qualitative analysis of primary and secondary spectra data from mass spectrometry assays was performed. The metabolite quantification was done by using multiple reaction monitoring (MRM) mode of triple quadrupole mass spectrometry. After obtaining the metabolite spectra of different samples, the peak areas of the mass spectra of all substances were integrated, and the integration of the mass spectra of the same metabolite in different samples was corrected (Fraga et al., 2010).

The metabolites of the samples were analyzed qualitatively and quantitatively by mass spectrometry through the software analyst 1.6.3 which opens the browsing of the downstream raw data after the mass spectrometry analysis. The MRM metabolite detection multi-peak map showed the substances that could be detected in the samples, with each differently colored mass spectrometry peak representing one metabolite detected. The signal intensity (CPS) of the characteristic ions was obtained in the detector by screening each substance with a triple quadrupole, and the sample offline mass spectrometry file was opened with MultiaQuant software to perform peak integration and calibration work. A quality control sample was inserted in every 10 samples analyzed to monitor the reproducibility of the analysis process.

Fold Change (Fold Change) represented the ratio of expression between two samples (groups), and metabolites with fold change ≥ 2 and fold change ≤ 0.5 were selected as the final differential metabolites.

### Transcriptome sequencing

Young poplar stems treated at different temperatures for different time periods were collected, rapidly frozen in liquid nitrogen, and later stored in an ultra-low temperature incubator at -80°C. Total RNA was extracted using a plant RNA extraction kit (Takara, Dalian, China) as described, while RNA concentration and purity were measured by NanoDrop 2000 (Thermo Fisher Scientific, Wilmington, DE, United States). RNA integrity was assessed using the RNA Nano 6000 assay kit from the Agilent Bioanalyzer 2,100 system (Agilent Technologies, CA, United States). mRNA-seq library was created by mRNA-Seq Sample Preparation Kit (Cat # RS-930–1,001, Illumina Inc., San Diego, CA).

The RNA seq was performed on paired-end and data analysis was performed as follows (Cong, 2011). The raw data was processed in fast format by an internal script. In this process, clean reads were obtained by removing reads containing adapters, reads containing ploy-N, and low-quality reads of the raw data. At the same time, Q20, Q30, GC-content and sequence repeat levels of clean reads were calculated. These clean reads were then mapped to the reference genome sequence. On the basis of the reference genome, only the exact matches or mismatched reads were further analyzed and annotated. The reference genome was localized using Hisat2 software.

Gene expression levels were estimated by transcribed fragments per kilobase per million (FKPM) fragments. Differential expression analysis of the two samples was performed using R edge. The FDR < 0.01 & Fold Change ≥2 was used as the threshold for significant differences in expression. Gene functions were annotated based on the following databases: KOG/COG (Homology Protein Cluster); KO (KEGG Ortholog database); Nr (NCBI non-redundant protein sequences); Nt (NCBI non-redundant nucleotide sequences); (containing protein families); Swiss-Prot (a manually annotated and reviewed protein sequence database); (gene ontology). Gene Ontology (GO) enrichment analysis of differentially expressed genes (DEGs) was performed using GO seq R package based on the Wallenius non-central hypergeometric distribution (Young et al., 2010). KOBAS software was used to detect statistical enrichment of differentially expressed genes in KEGG pathway. P. trichocarpa V3.0 was used as the reference genome (https://phytozome.jgi.doe.gov/pz/portal.html).

### RT-qPCR (reverse transcription-quantitative real-time polymerase chain reaction) assays

The transcript levels of *PtrMYBs*, *PtrCCR2* and *PtrC3'H1* were examined by RT-qPCR. *PtrACTIN* was used as the control. The execution was performed through SYBR Green Fluorescence method described in a previous article (Zhao et al., 2020). The cycling conditions for RT-qPCR were as follows: pre-denaturation at 95°C for 10 min, denaturation at 95°C for 10 s, annealing at 60°C for 10 s, and extension at 72°C for 30 s for a total of 40 cycles. Fluorescence signals were obtained at the extension phase of each cycle. Data analysis was performed by the 2-ΔΔCt method. The primers used in this experiment were shown in Supplementary Table S1.

## Results

### Changes of lignin content in poplar stem caused by high temperature

**FIGURE 1:**
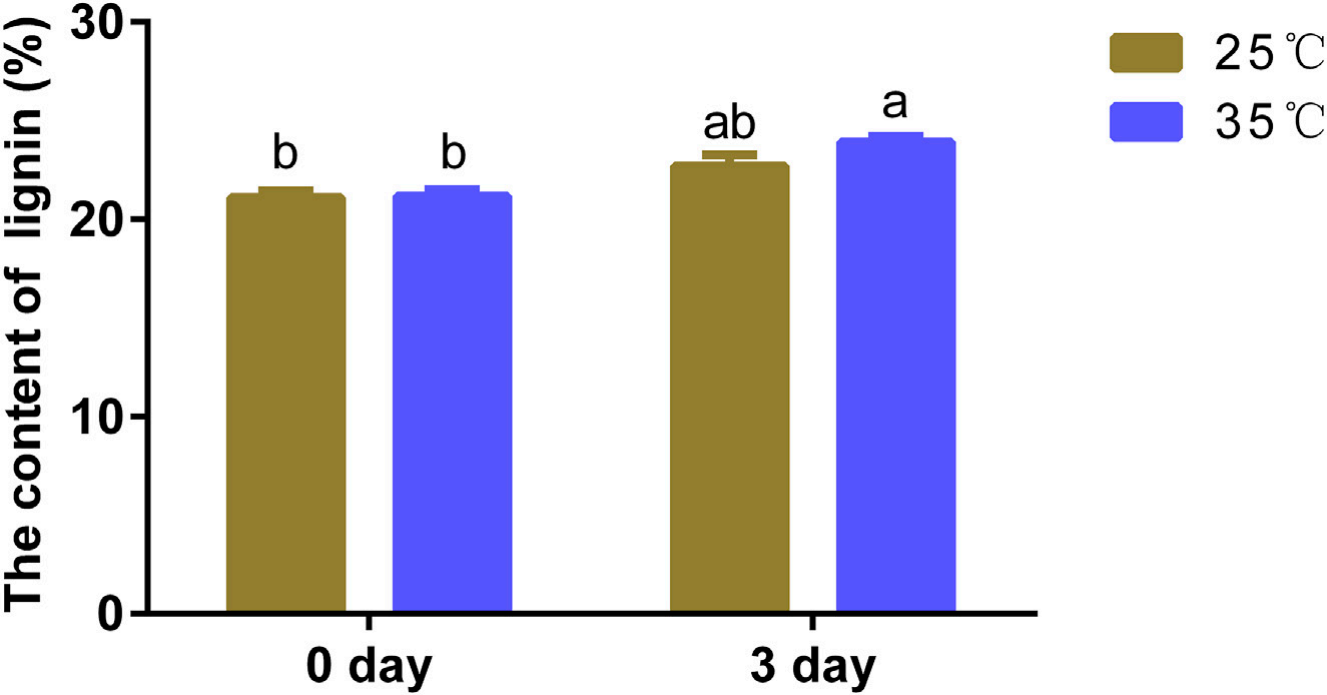
High temperature changed the lignin content in poplar. Lignin content of poplar stem after treatment of 35°C (heat treatment) and 25°C (normal treatment). The same letter of *P*_*0.05*_ level represents the no difference while different *P*_*0.05*_ level showed the imparity. The data showed as the mean ± SE was repeated three times. a and b represent significant differences, while ab and a or ab and b are not significantly different in both pair because they have duplicate letters.

The biosynthesis of lignin can be altered by kinds of abiotic and biotic stresses (Moura et al., 2010; Behr et al., 2019). To investigate the effect of high temperature on lignin content of poplar, poplar “84k” of 40 days old tissue culture plantlets were treated with 35°C high temperature and 25°C normal temperature. Compared to the plantlets grown at normal temperature of 25°C, determination of lignin content showed a slight increase in lignin content when exposed to high temperatures (Figure 1). The results showed that poplar tissue culture plantlets tended to accumulate more lignin to resist heat stress when exposed to high temperature.

### Effect of high temperature treatment on metabolites in lignin synthesis pathway

**FIGURE 2:**
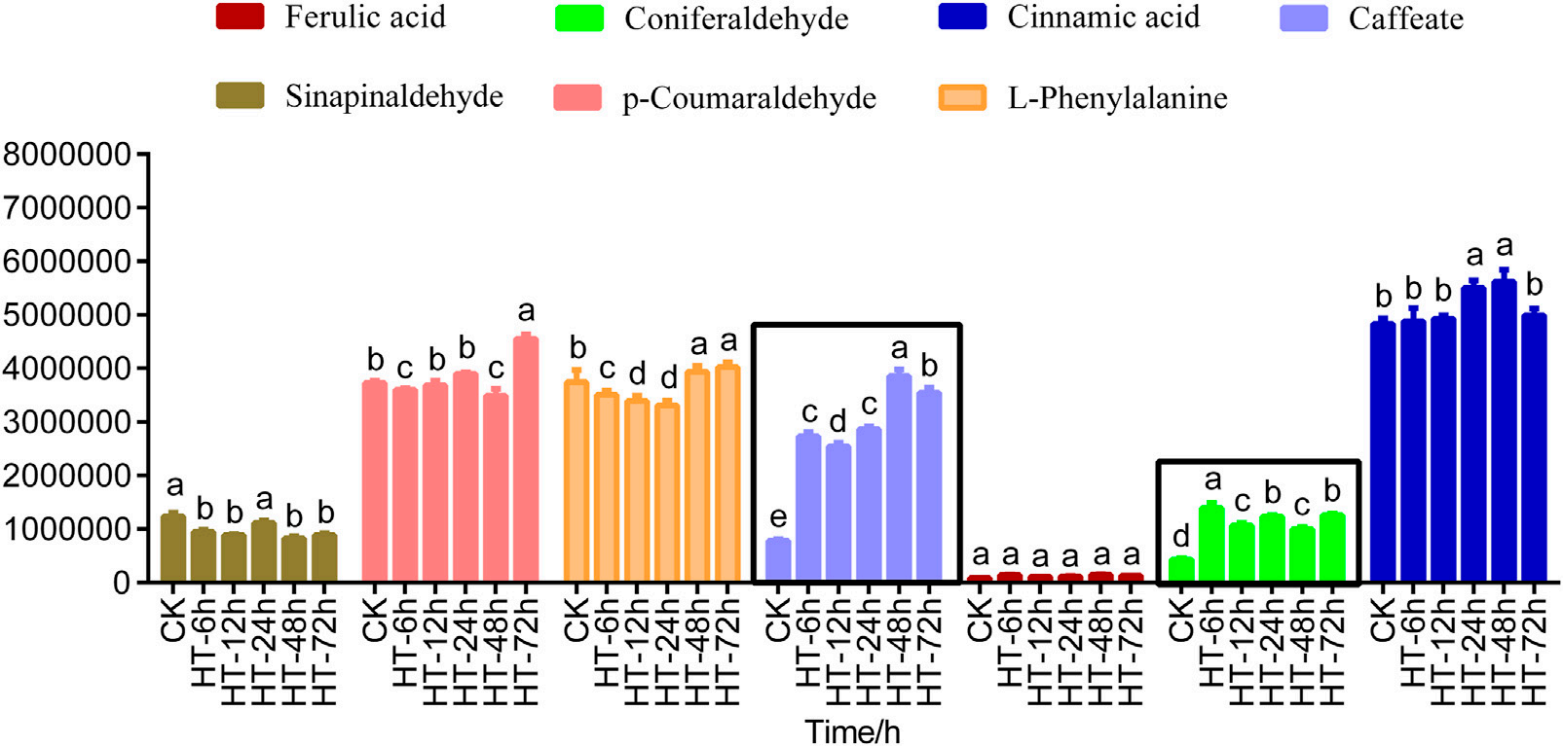
Analysis of intermediates in lignin pathway. Histogram of lignin level of 0 (CK), 6, 12, 24, 48, 72 h after treated with heat stimulus. The data showed as the mean ± SE was repeated three times. HT means high temperature. The same letter of *P*_*0.05*_ level represents the no difference while different *P*_*0.05*_ level showed the imparity.

In order to further explore the substance in lignin synthesis pathway, targeted metabolomics analysis of 14 metabolites in the lignin synthesis pathway was performed. Lignin metabolism intermediates such as sinapic acid, coniferyl alcohol, p-coumaric acid, sinapyl alcohol, p-coumaryl alcohol, caffeyl alcohol, caffeyl aldehyde were detected but could not be quantified and analyzed because of their low levels. Among the seven intermediates of lignin metabolism quantified, sinapinaldehyde, p-coumaraldehyde, l-phenylalanine, ferulic acid and cinnamic acid showed no significant changes at different time periods after high temperature treatment (Supplementary Material S1; Figure 2). However, it was found that caffeate and coniferous aldehyde always showed an upward trend in different periods after high temperature treatment, which may be the main reason for the increase of lignin content in poplar under high temperature stress (Supplementary Material S1; Figure 2).

### Trait of transcriptional database libraries

Both TFs and structural genes are involved in lignin synthesis by affecting the relevant enzymes in the lignin pathway. In order to investigate which genes are involved in the regulation of lignin after high temperature stress, 18 cDNA libraries (6 samples, 3 replicates) from 35°C treated poplar stems at different time periods were established. 892458006 high-quality reads were obtained and transcriptome data were stored in NCBI database with SRA accession number PRJNA742770. About 70.69–74.14% reads were matched to the genome. It was found that the percentage (about 68.25–71.80%) of unique reads and clean reads in the genome was not much different. Therefore, the data quality and quantity of the transcriptome were reliable (Supplementary Material S2).

### Characterization of differential genes expression profiles of poplar stem in response to high temperature

**FIGURE 3:**
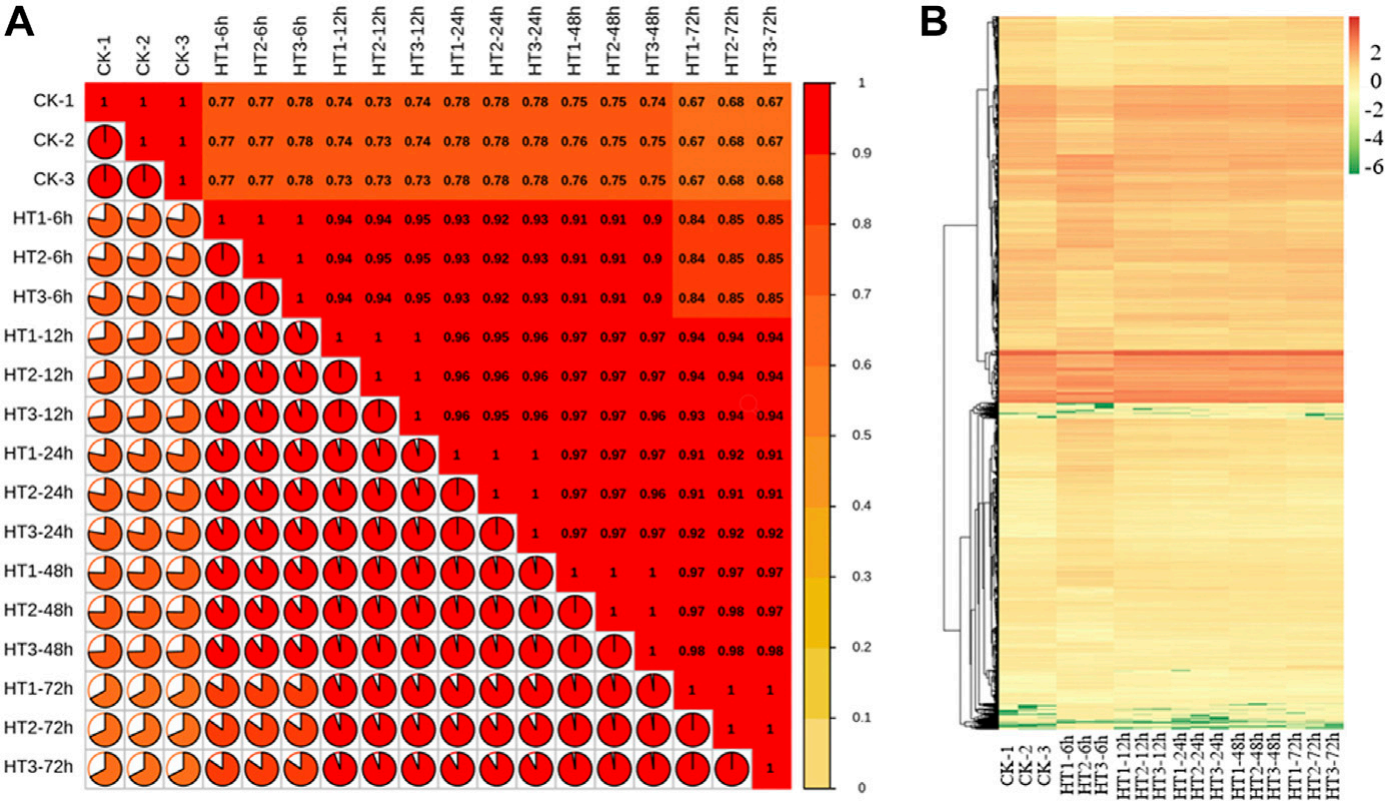
Analysis of gene expression in response to high temperature. **(A)** Heat map of correlation coefficient values across samples based on RNAseq FPKM. **(B)** Cluster analysis of DEGs of 0 (CK), 6, 12, 24, 48, 72 h after treated with high temperature treatment. CK is the sample of 0 h. HT means high temperature. DEG means differential expressed genes.

Five two-by-two comparisons, including 0 h versus 6, 12, 24, 48, 72 h respectively, were used to analyze the expression profiles of differential genes in the response to high temperature treatment in poplar stems. By correlating FPKM values, the FPKM heat map was constructed. The correlation value of three repeat samples at the same time was 1 while the correlation values of heat maps at normal and heat temperature at different period were all less than 0.8, indicating a high degree of consistency in biological repetition with high temperature treatment (Figure 3A).

The genes with log2 foldchange ≥1 and FDR ≤0.05 were significant DEGs. According to the analysis of transcriptome data, there were 6,480, 2,421, 1,640, 1,452, 778 genes changed at 6, 12, 24, 48, and 72 h, respectively (Supplementary Material S3; Figure 3B). In detail, compared with 0 h, the transcription level of 3,274, 1,446, 939, 852, 534 genes increased respectively, while the transcription level of 3,206, 975, 703, 600, 244 genes decreased after 6, 12, 24, 48, and 72 h high temperature treatment (Supplementary Material S3; Figure 3B). The studies showed that high temperature can stimulate the expression of related genes in poplar stem, and the gene change caused by high temperature reduced with time.

### Functional analysis of differential expressed genes (DEG)

To further study the comprehensive annotation of DEGs in response to heat stress, the COG, KOG, GO and KEGG, protein databases were applied to annotate all the DEGs.

**FIGURE 4:**
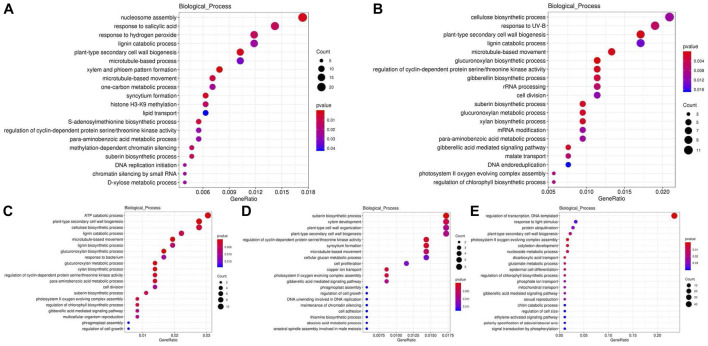
GO enrichment analysis of DEG at 6 **(A)**, 12 **(B)**, 24 **(C)**, 48 **(D)**, 72 **(E)** hours compared with the control group (0 hour) after high temperature treatment. CK is the sample of 0 h. HT means high temperature. DEG means differential expressed genes.

Twenty-six functional clusters were used for COG analysis. The COG analysis varies with the processing time. In general, both COG and KOG analysis revealed that the differential genes were mainly involved in inorganic ion transport and metabolism, carbohydrate transport and metabolism, energy production and conversion, signal transduction mechanisms, RNA processing and modification, posttranslational modification, protein turnover, chaperones, general function prediction only, cell wall/membrane/envelope biogenesis and other pathways (Supplementary Material S4, S5). As can be seen from the results, the pathways involved in the protein data analysis of KOG and COG are almost identical. In addition, the changes of genes related to secondary metabolites biosynthesis, transport and catabolism suggest that some secondary metabolites may also participate in the response of poplar stems to high temperature. This indicated that poplar responds to the adverse effects of high temperature in a variety of ways, including energy conversion and signal transduction. For GO enrichment analysis, three terms including molecular function, biological process and cellular component were used to classify the DEGs function. Among all DEGs, 3,319 genes were compared to the GO enrichment data. Of which, 1,610, 665, 466, 423, and 228 genes changed after high temperature treatment of 6, 12, 24, 48, and 72 h, respectively. The changed genes were mainly related to cell, organelle, cell part, cell junction, and membrane in cellular component; catalytic activity and binding in molecular function; and metabolic process, cellular process, single-organism process, response to stimulus, biological regulation in biological process (Supplementary Material S6). By analyzing the biological process, it was found that genes were related to plant-type secondary cell wall biogenesis, the lignin catabolic process and lignin biosynthetic process which may influence lignin content changed significantly (Figure 4).

Differential genes were selected for annotation analysis of KEGG database after high temperature treatment at 6, 12, 24, 48 and 72 h. The results showed that the differential gene enrichment pathway decreased with the extension of treatment time (Supplementary Material S7). KEGG enrichment analysis at 0 h vs. 6, 12, 24, 48, 72 h demonstrated that the enrichment pathways were mainly involved in starch and sucrose metabolism, biosynthesis of amino acids, plant hormone signal transduction, carbon metabolism, phenylpropanoid biosynthesis, etc (Supplementary Material S7). In general, the KEGG enrichment analysis of phenylpropanoid biosynthesis may be related with lignin synthesis.

### Lignin synthesis related genes responding to high temperature stress

To investigate the genes involved in lignin synthesis in response to high temperatures, MYB, NAC and some structural genes in lignin synthesis pathway were analyzed. The results showed that a total of 843 genes may be involved in the process of lignin synthesis in poplar in response to high temperatures. In detail, there were 9 NACs, 558 MYBs, 6 *PALs*, 1 *C4H*, 2 *C3'Hs*, 16 *4CLs*, 35 *HCTs*, 32 *CCRs*, 1 *COMT*, 3 *F5Hs*, 4 *CCoAOMTs*, 42 *CADs*, 46 *LACs*, and 88 *PODs* (Supplementary Material S8).

**FIGURE 5:**
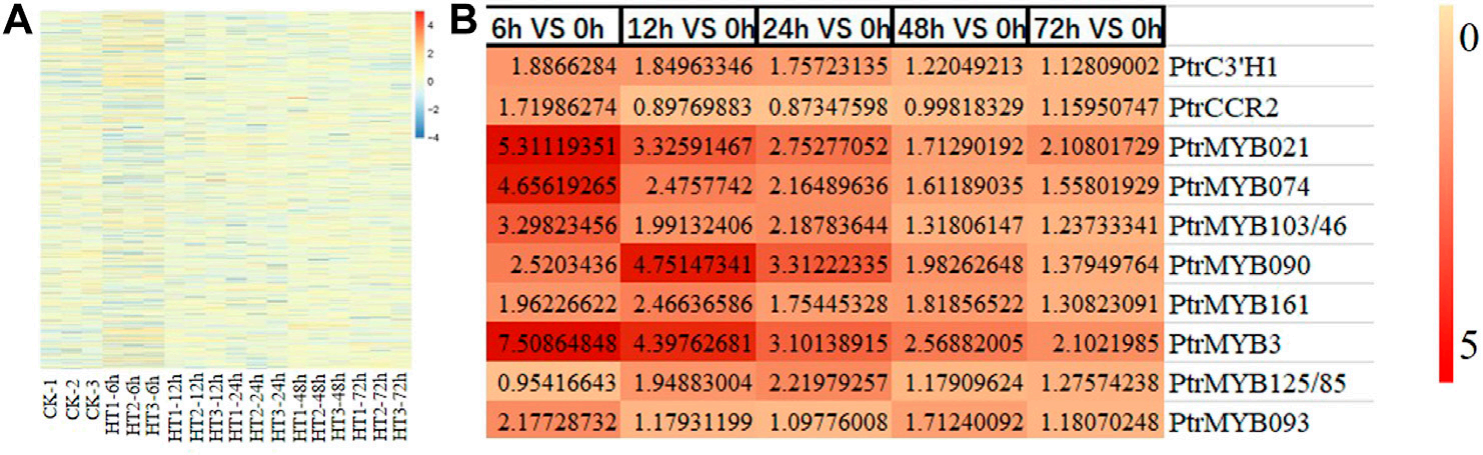
The variation trend of lignin-related genes induced by heat stress. **(A)** Cluster analysis of lignin-related genes induced by heat stress. **(B)** The expression level of some lignin-related genes at different time periods (6, 12, 24, 48, 72 h) compared with the control group (0 h) after HT treatment. CK is the sample of 0 h. HT means high temperature. DEG means differential expressed genes.

The trends of the above lignin-related genes were analyzed by RPKM values (Figure 5). Among the exchanged genes in lignin synthesis, *PtrC3'H1* (Potri.006G033300), *PtrCCR2* (Potri.003G181400), *PtrMYB021* (Potri.009G053900) and *PtrMYB074* (Potri.015G082700), *PtrMYB103/46* (Potri.003G132000), *PtrMYB090* (Potri.015G033600), *PtrMYB161* (Potri.007G134500), *PtrMYB3* (Potri.001G267300), *PtrMYB125/85* (Potri.003G114100), *PtrMYB093* (Potri.004G138000) may be involved in high temperature-induced synthesis of caffeate, coniferaldehyde and lignin. The other genes with more significant changes might also be involved in the process of lignin synthesis caused by high temperature.

### RT-qPCR analysis of major lignin synthesis related genes

**FIGURE 6:**
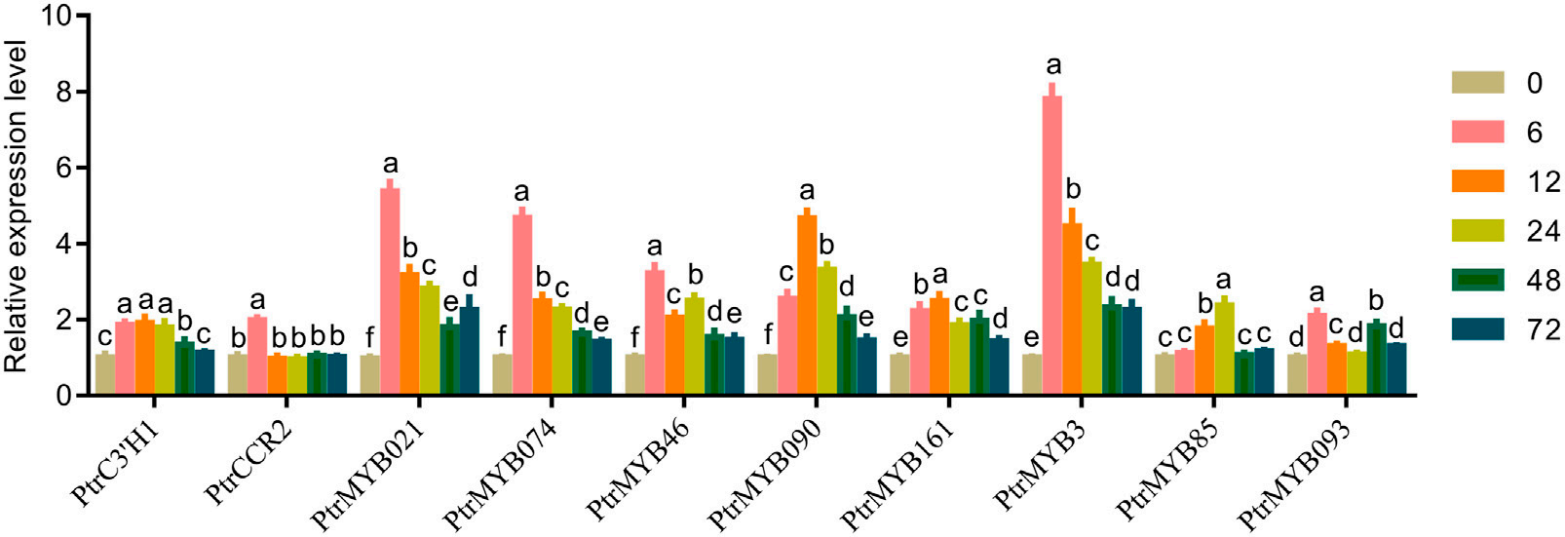
RT-qPCR analysis of lignin-related genes. RT-qPCR analysis of *PtrC3'H1* (Potri.006G033300), *PtrCCR2* (Potri.003G181400), PtrMYB021 (Potri.009G053900) and PtrMYB074 (Potri.015G082700), PtrMYB103/46 (Potri.003G132000), PtrMYB090 (Potri.015G033600), PtrMYB161 (Potri.007G134500), PtrMYB3 (Potri.001G267300), PtrMYB125/85 (Potri.003G114100), PtrMYB093 (Potri.004G138000) of poplar stem with high temperature treatment for 0, 6, 12, 24, 48 and 72 h. The same letter of *P*_*0.05*_ level represents the no difference while different *P*_*0.05*_ level showed the imparity. The data showed as the mean ± SE was repeated three times. between.

According to the network map of lignin synthesis pathway, some TFs such as MYBs, NACs and structural genes were found to be involved in the synthesis of caffeate, coniferaldehyde and lignin in response to high temperatures in poplar. By searching for related genes and designing primers, the transcript levels of several important genes, *PtrC3'H1* (Potri.006G033300), *PtrCCR2* (Potri.003G181400), PtrMYB021 (Potri.009G053900) and PtrMYB074 (Potri.015G082700), PtrMYB103/46 (Potri.003G132000), PtrMYB090 (Potri.015G033600), PtrMYB161 (Potri.007G134500), PtrMYB3 (Potri.001G267300), PtrMYB125/85 (Potri.003G114100), PtrMYB093 (Potri.004G138000), were examined by RT-qPCR analysis. The results showed that the transcript levels of these genes and the data from transcriptome analysis were consistent (Figure 6). It indicated that the transcriptome data were reliable and reproducible, and that these genes may play important roles in the regulation of lignin synthesis in response to high temperature in poplar seedlings.

## Discussion

### The lignin content increased under high temperature stress

High temperature stress is one of the major environmental factors that affect plant distribution and development and limit plant resource utilization (Zhu, 2016). Because of a series of advantages such as small genome, rapid growth and high economic value, poplar is a model for studying forest plants and also suitable for transcriptome study (Taylor, 2002). In this study, poplar tissue culture plantlets “84K” was used as the research material, and treated at 35°C for 6, 12, 24, 48, and 72 h, it was found that the lignin content in the stem of poplar tissue culture seedlings increased significantly after high temperature treatment (Figure 1). Lignin, a class of phenylpropane polymers, is essential for plant growth and development and in response to biotic and abiotic stress stimuli (Cesarino, 2019). Once the stem of a plant is exposed to high temperatures, the cell walls rapidly lignify (Crivellaro and Büntgen, 2020). Lignin accumulates and is deposited in the cell wall, strengthening the stiffness of the cell wall, making the xylem cell wall less permeable to water and which facilitates the long-distance transport of water, minerals and organic matter in plants (Zhao, 2016). When plants are subjected to high temperature stress, they will enhance their resistance to stress by synthesizing more lignin.

### The caffeate and coniferaldehyde may be the key factor for the increase of lignin content of poplar stem responding to high temperature

Lignin synthesis involves the production of many intermediate metabolites (Boerjan et al., 2003; Pu et al., 2009; Barros et al., 2015). It was found that intermediates and their secondary metabolites produced by phenylpropanoid metabolic pathway not only improved disease resistance but also regulated and promoted plant resistance to abiotic adversities such as low temperature, high temperature, and UV radiation (Dixon and Paiva, 1995). Under abiotic stresses such as ozone and drought, the content of phenylpropanoid metabolic secondary organisms including gallic acid, caffeic acid, and coumaric acid increased (Dixon and Paiva, 1995; Sgarbi et al., 2003; Xu et al., 2022). Similarly, our study found that high temperature promoted the accumulation of caffeic acid. Caffeic acid is a key intermediate in the lignin metabolic pathway and is ubiquitous in the plant kingdom (Deng and Lu, 2017; Reuter et al., 2020). In addition, mechanical injury, ethylene and methyl jasmonate treatment caused changes of caffeic acid (Reuter et al., 2020). The increase of caffeic acid content caused an increase of lignin content (Bubna et al., 2011). Therefore, it is possible that the elevation of caffeic acid stressed by high temperature is the cause of lignin elevation.

In addition to the elevated caffeic acid content, the coniferaldehyde content also increased after high temperature treatment. Coniferaldehyde is a key intermediate metabolite in lignin synthesis and sometimes functions as a lignin monomer to influence lignin synthesis (Lapierre et al., 2004; Anderson et al., 2015). Coniferaldehyde also modulates the lignin metabolic pathway by altering the content and composition of lignin, including through the conversion to ferulic acid and its derivatives (Van Acker et al., 2017). Coniferaldehyde content of lignin and other components filled in the cell wall can effectively inhibit the degradation of the maize cell wall (Grabber et al., 1998). In addition, physical, biochemical and mechanical properties of lignin altered because of the amount of coniferaldehyde residues (Fornalé et al., 2012; Van Acker et al., 2013; Wang et al., 2018; Blaschek et al., 2020). In a word, the synthesis of these substances may be an important reason for the elevated lignin content caused by high temperature.

### The possible genes involved in lignin synthesis of poplar stem responding to high temperature stress

The synthesis of caffeic acid is regulated by several genes. P-coumarate to caffeate reactions are mainly catalyzed by C3'H (Mottiar et al., 2016). C3'H belongs to cytochrome P450 class. Schoch et al. cloned and isolated the C3'H gene from cytochrome P450 of *Arabidopsis* genome (Schoch et al., 2001). Numerous studies have shown that C3'H is the rate-limiting enzyme of phenylpropanoid pathway in the lignin biosynthetic pathway, which can catalyze the C3 hydroxylation reaction on the benzene ring of the phenylpropanoid structure and can determine the carbon source flow of lignin monomers. Down-regulation of *C3'H* expression can both reduce lignin content and change monomer composition, which can reduce the cost of plant papermaking (Abdulrazzak et al., 2006). In hybrid poplar (*Populus grandidentataalba*), lignin monomer composition and lignin content were significantly reduced in *C3'H* RNAi strains (Coleman et al., 2008). Among the two *C3'H* genes subjected to high temperature-stimulated expression changes in this study, *PtrC3'H1* (Potri.006G033300) had a very high similarity with *C3'H* (GenBank accession no. EU391631) and its transcript levels were significantly elevated by high temperature stress. This suggested that *PtrC3'H1* (Potri.006G033300) may be involved in caffeic acid production and thus affect lignin content after high temperature induction.

There are two directions regarding the destination of caffeic acid, one is the synthesis of ferulic acid in the presence of COMT (caffeic acid O-methyltransferase); the other is the generation of caffeoyl-CoA in the presence of 4CL (4-coumarate: CoA ligase) (Mottiar et al., 2016). In hybrid poplar (*Populus trichocarpa* and *Populus deltoides*), overexpression of *Pto4CL* gene was effective in increasing lignin content (Allina et al., 1998). The *Pt4CL1* gene was specifically expressed in organs with high lignin content such as xylem and was involved in the lignin synthesis process in aspen (Hu et al., 1998; Naik et al., 2018). Among the 16 high temperature-induced *4CLs*, the transcript expression levels of two genes, (Potri.004G102000) and (Potri.017G112800), were consistently reduced by high temperature stimulation, suggesting that the reduction in their transcript levels may have influenced the elevation of caffeic acid. In this study, it was found that only one *PtrCOMT* (Potri.006G120000) altered with high temperature treatment and its transcript level was reduced only at 6 h. This may also be one of the reasons for the elevated caffeic acid content.

Coniferaldehyde synthesis is influenced by several genes. Among them, CCR and COMT have the ability to promote the production of coniferaldehyde, while F5H and CAD make coniferaldehyde to produce other substances. It was found that 32 *CCRs*, 1 *COMT*, 3 *F5Hs* and 42 *CADs* were induced by high temperature in this study. Among them, *PtrCOMT* (Potri.006G120000), whose expression was reduced by high temperature induction, was not responsible for the elevation of coniferaldehyde. Lignin contents in down-regulated transgenic strains of *CCR1* gene were significantly lower compared to wild type (Van Acker et al., 2014). 12 of 32 *CCRs* induced by high temperature significantly elevated genes may be involved in high temperature-induced coniferaldehyde and lignin synthesis. *PtrCCR2* (Potri.003G181400) was related with lignin content (De Meester et al., 2020). With our results, *PtrCCR2* (Potri.003G181400) exhibited evidently high expression after heat induction. Besides, two genes (Potri.005G117500, Potri.007G016400) were up-regulated by high temperature induction in three *F5Hs*, indicating that they are not factors in coniferaldehyde elevation. However, there was no significant reduction in *PtrCADs* transcription levels among the numerous heat-induced *PtrCADs*, suggesting that *PtrCADs* may be not responsible for the elevation of coniferaldehyde.

### Analysis of TFs that may affect lignin synthesis in response to high temperatures

Lignin synthesis is also regulated by TFs. The NAC MYB-based gene regulatory network (NAC-MYB-GRN) is widely considered to be the main pathway regulating lignin synthesis (Ohtani and Demura, 2019). NAC is the primary network controlling lignin synthesis (Ohtani and Demura, 2019). The secondary regulatory network are some MYB TFs such as AtMYB46 and AtMYB83 which are the downstream of NAC TFs (Ohtani and Demura, 2019). NAC and MYB affect lignin synthesis by regulating the transcription levels of some structural genes that are critical in the process of lignin synthesis.

NAC TFs, especially VND6 and SND1, are thought to be the master regulators of SCWs (Zhong et al., 2007; Lin et al., 2017). In poplar, PtrVND6 and PtrSND1 can participate in the synthesis of lignin as well as cellulose, hemicelluloses by affecting the differentiation of secondary cell walls (SCWs) (Lin et al., 2017). Overexpression of *VND6* and *SND1* caused impaired lignin content and unhealthy plant development (Lin et al., 2017). However, among the nine NACs identified that were induced by high temperature in this study, the changes were not obvious, and none of them were reported to be involved in lignin synthesis or SCWs formation. Some MYB TFs acted as the downstream of NAC TFs and are involved in lignin synthesis by directly binding to the promoters of lignin structural genes (Behr et al., 2019). PtrMYB021 and PtrMYB074 regulated the growth and development of poplar by participating in lignin production (Chen et al., 2019). In addition, several other MYB TFs were found to be involved in the lignin metabolic pathway (Chen et al., 2019). By analyzing 558 MYB TFs induced by high temperature, it was found that the transcript levels of *PtrMYB021* and *PtrMYB074* were significantly elevated by high temperature induction. MYB46 and MYB83 were considered to be a secondary master switch of SND1 involved in lignin synthesis (Zhong et al., 2007; Ko et al., 2014). PtrMYB103/46 (Potri.003G132000), a homolog of AtMYB46, was also induced by high temperature although weakly. Some other MYB TFs such as PtrMYB002, PtrMYB003, PtrMYB020, PtrMYB161, PtrMYB90, PtrMYB152, PtrMYB189, etc. have also been reported to be involved in lignin synthesis (Ko et al., 2014; Chen et al., 2019). Transcriptome data showed that PtrMYB090 (Potri.015G033600), PtrMYB161 (Potri.007G134500), PtrMYB3 (Potri.001G267300), PtrMYB125/85 (Potri.003G114100), PtrMYB093 (Potri.004G138000) transcript expression levels were elevated by high temperature induction. These PtrMYBs may play an important role in lignin accumulation of poplar stem in response to high temperature.

## Conclusion

**FIGURE 7:**
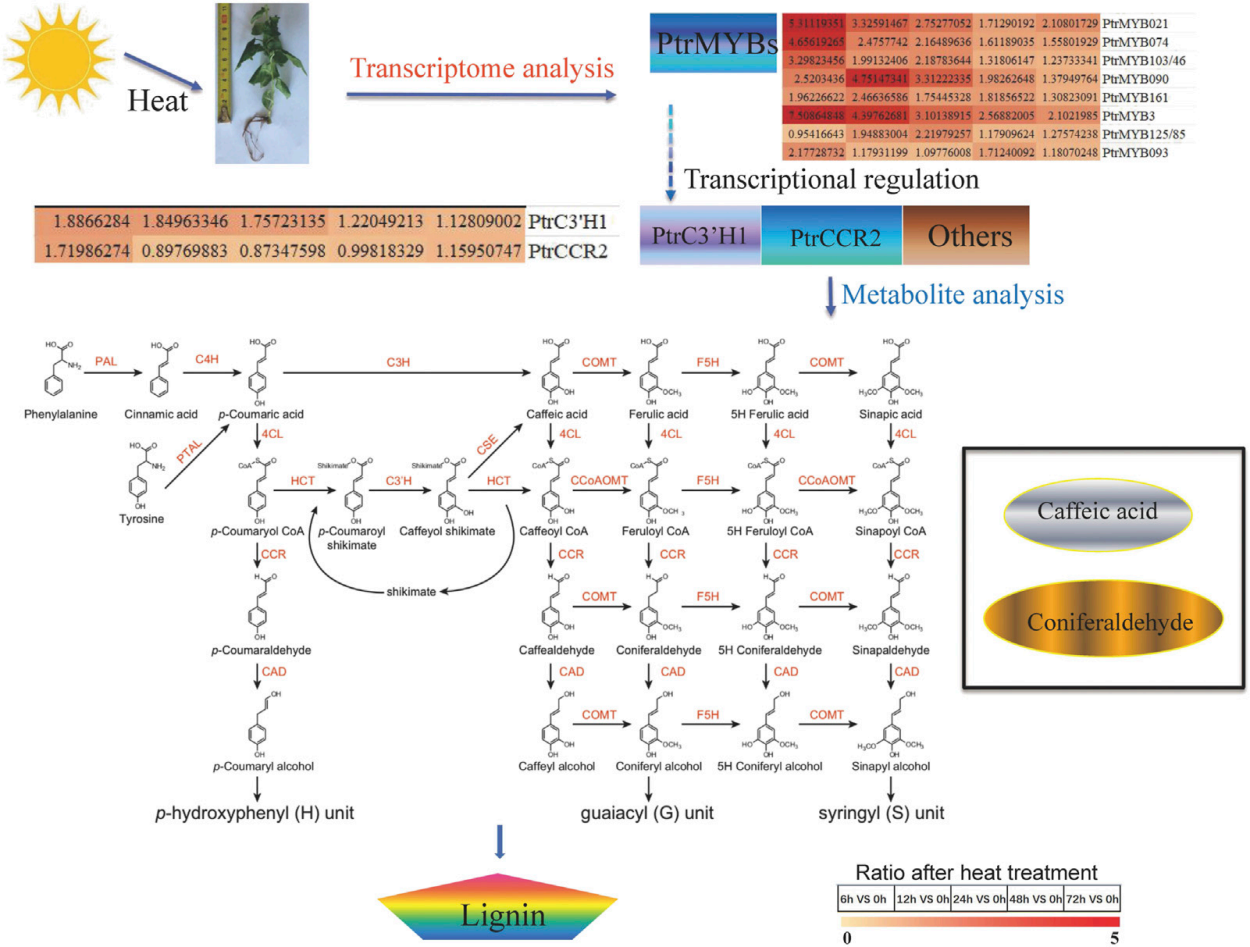
Regulatory mechanism between high temperature and lignin content. High temperature brings about the increased lignin content. Transcriptome analysis indicated that some vital transcription factors such as PtrMYBs, others lignin related transcription factors and lignin-related structural gene such as *PtrC3’H1*, *PtrCCR2* were induced by high temperature. *PtrC3’H1* may be a possible factor leading to the decrease of caffeate content, while *PtrCCR2* may bring about the lessen of coniferaldehyde. caffeate, coniferaldehyde represents the different lignin precursors.

In general, when subjected to high temperature stress, poplar produces lignin through secondary metabolic pathways to resist adverse abiotic stresses. Several TFs (NAC, MYB) and structural genes (*PAL*, *C4H*, *C3'H*, *4CL*, *HCT*, *CCR*, *COMT*, *F5H*, *CCoAOMT*, *CAD*, *LAC*, and *POD*) in lignin synthesis pathway participated in the high temperature response of poplar (Figure 7; Zhang et al., 2020). In addition, the intermediates of lignin metabolic pathway (caffeate and coniferaldehyde) were also affected by high temperature and thus regulated lignin content. High temperature increases lignin contents of poplar stem may via inducing the PtrCCR2-participated biosynthesis caffeate and coniferaldehyde possibly under the regulation of PtrMYB021 and PtrMYB074 or some other MYB TFs. The related molecular mechanism needs further analysis and verification. Besides, the changes of H, G and S types of lignins and the changes of lignin ratios/proportions will be research emphases on molecular mechanism analysis. Identification of the key substances and genes in the lignin pathway in response to high temperature is of great significance to explore the high temperature resistance of poplar.

## Data Availability

The data presented in the study are deposited in the BioProjectin NCBI repository (https://www.ncbi.nlm.nih.gov/bioproject/?term=PRJNA742770), accession number is PRJNA742770.
