# Supplementary material for: High temperature increased lignin contents of poplar (Populus spp) stem via inducing the synthesis caffeate and coniferaldehyde
Source: Front Genet. 2022 Sep 9;13:1007513. doi: 10.3389/fgene.2022.1007513 (PMC9500204; doi:10.3389/fgene.2022.1007513)
Supplement: Supplementary file 5 [file Table1.DOC]

**High temperature induces caffeate and coniferaldehyde contributed to lignin synthesis in poplar**

**Supplemental Table 1: List of primers used in this research.**

| Gene name | Primer sequence (5'→3') |
| --- | --- |
| PtrMYB021 | F: GCATTTTCACCCCAAGAAGA  R: CCGCAATTTGAGACCACCTA |
| PtrMYB074 | F: TTTTGGGTAACAGGTGGGCT  R: CTTCTCCTCTTTCACCCCCAC |
| PtrC3’H1 | F: GTGTCATGACCGAGGCTGAT  R: AAGGGTTTCTTCCAGGTGGC |
| PtrCCR2 | F: GAACCCAGCTGATCCCAAGA  R: GATCCATGTACACAGCGCC |
| PtrMYB3 | F: GTGGTCACAGGCAACCAATTT  R: TACTCTCGAGTGGCGGAAGA |
| PtrMYB161 | F: TCTAGCAGAGGCCATTGGAG  R: ACAACTCTTCCCCTGATC |
| PtrMYB125/85 | F: CCAAGCCTGAGGAAAGCTCA  R: TCTGCGGCCAGAGGAATAAT |
| PtrMYB103/46 | F: ACATGGAGAACATGGTGCCT  R: GTAGCAGCTCCCATACTTGATGA |
| PtrMYB093 | F: GCAGATCTCCTTGCTGTGAG  R: TCTAAGCAAACCAGCAGCCTT |
| PtrMYB90 | F: ATTGGAGGCCTGCAGAAGAT  R: AACCTACAACTCTTCCCTGATC |
| PtrACTIN | F: ACACGGGGAGGTAGTGACAA  R: CCTCCAATGGATCCTCGTTA |
